# Defining a target product profile for antimicrobials to treat severe paediatric bacterial enteric infections in low- and medium-income countries, caused by *E. coli*, *Shigella*, *Salmonella* and *Campylobacter* species

**DOI:** 10.1093/jacamr/dlag128

**Published:** 2026-07-10

**Authors:** Marie Attwood, Tamara Choola, Erin Duffy, Valeria Gigante, Joseph Harwell, Lianne A Hulshof, Samuel M Kariuki, Derry K Mercer, Kristina M Orrling, David Paterson, Dorothy Yeboah-Manu, Alasdair MacGowan

**Affiliations:** Bristol Centre for Antimicrobial Research & Evaluation (BCARE), North Bristol NHS Trust, Bristol, UK; Afrocab, Treatment Access Partnership (AFROCAB), Lusaka, Zambia; CARB-X, Boston University, Boston, MA 02215, USA; AMR Department, World Health Organization (WHO), Geneva, Switzerland; Senior Clinical Director, Clinton Health Access Initiative, Dorchester Avenue, Boston, MA, USA; Global Health Innovation, Lygature, Jaarbeursplein 6, Utrecht 3521 AL, the Netherlands; Drugs for neglected diseases initiative, Kenya Medical Research Institute, Nairobi, Kenya; Institute for Technological Innovation in Microbiology, BIOASTER, 40 avenue Tony Garnier, Lyon 69007, France; INCATE, University of Basel, Petersgraben 35, Basel 4051, Switzerland; Global Health Innovation, Lygature, Jaarbeursplein 6, Utrecht 3521 AL, the Netherlands; ADVANCE-ID, National University of Singapore, Lee kong Chian Wing 119077, Singapore; Noguchi Memorial Institute for Medical Research, University of Ghana, Ghana, Legon; Bristol Centre for Antimicrobial Research & Evaluation (BCARE), North Bristol NHS Trust, Bristol, UK

## Abstract

Paediatric diarrhoea is associated with 500 000 childhood deaths each year in low- and middle-income countries (LMICs). Enteropathogenic *Escherichia coli*, *Shigella* spp., *Salmonella* spp. and *Campylobacter* spp. are among the most common causative bacterial pathogens, however, their burden and rates of antimicrobial resistance (AMR) are poorly quantified. Current treatment typically involves oral and intravenous rehydration fluids, nutritional support for malnourished children, antibiotics for severe bacterial infections, antiemetic and anti-diarrheal medications, and analgesics. The World Health Organization recommends first-line empirical treatment of ciprofloxacin, with alternatives of third-generation cephalosporins or azithromycin. At present, no new antibacterials are in development specifically to treat paediatric diarrhoea.

Target product profiles (TPPs) are used typically in drug development and describe the desired characteristics of a new therapeutic. In this paper, we aim to define a TPP for the development of novel antimicrobials for the treatment of severe paediatric diarrhoea in LMICs. Oral and intravenous formulations are preferable. Safety, tolerability, resolution of clinical signs and symptoms must be equivalent or better than standard of care (SoC) therapy. Interactions with commonly used therapies such as zinc, milk and traditional cures should be minimal. In addition, formulations should be stable at elevated temperatures and for extended periods of time to facilitate access and distribution across LMICs.

## Introduction

Severe paediatric diarrhoea, of which the most common causative bacterial pathogens are *Escherichia coli*, *Shigella* spp., *Salmonella* spp. and *Campylobacter* spp. pose a significant public health burden. These pathogens disproportionately affect children between 6 months and 5 years of age, especially in low- and medium-income countries (LMICs) causing significant morbidity and mortality.^1^ According to the World Health Organization (WHO), enteric infections and diarrheal diseases are the second leading cause of death among young children in LMICs, killing >500 000 children under the age of 5 each year.^2,3^ UNICEF reports that children in LMICs who survive enteric infections are more prone to relapse due to the pathogen/s not being eliminated and/or reinfection due to inadequate water, sanitation and hygiene measures.^4^ Malnutrition within the paediatric population has been associated with an inability to resist opportunistic pathogens, altered immune responses, poor physical and/or cognitive development, stunting, delayed school readiness, reduced adult wages and generational poverty.^5–8^ There is a strong correlation between severe enteric infection and mortality, and an even stronger correlation between the development of sequelae and morbidity.^9^

Antimicrobial resistance (AMR) exacerbates the burden of enteric bacterial diseases. Overuse and misuse of antibiotics contribute to an increased prevalence of resistant strains, thereby reducing treatment options. AMR in enteric pathogens limits effective treatment, especially for vulnerable populations.^10^ Improved antibiotic stewardship, better sanitation and development of vaccines alone will not address this situation.^11^ New antimicrobial therapies are urgently needed to combat both the burden of enteric infections and the spread of AMR.

Target product profiles (TPPs) are integral tools in drug development, providing a framework for defining the essential characteristics of potential new therapies. Creating a comprehensive TPP is crucial for developing new treatments that meet both the medical needs of specific populations and the broader goals of society. This includes defining minimum and preferred targets for treatment populations, treatment characteristics and clinical criteria early in the process of drug development.

Understanding the clinical characteristics of paediatric diarrhoea, such as its burden, co-morbidities and other relevant health factors, is essential. Identifying the patient population is critical, and should be categorized by factors such as age, symptom severity and diagnostics. Essential clinical features of the infection must also be considered, including whether bacteraemia, fever, haematochezia and/or microbiological diagnosis of bacterial infection are evident. Other key considerations include the duration of therapy, mode of delivery, dosage form, treatment regimen, target efficacy, safety, pharmacokinetics and dynamics, cost profile and stability.

So far, there are no antibiotic therapies in development with the primary indication of enteric disease. However, there are numerous novel antibiotics in development for therapy of Gram-negative infections, which could be used in this patient population. Available vaccines include those for *Cholera* and Rotavirus infection, but there are still concerns about appropriate formulation for the target population.^12,13^ There are vaccines in development for enteric related pathogens such as; *Shigella*,^14^  *E. coli*,^15^  *Salomnella*^16^ and *Camplyobacter*,^17^ but they are not appropriate for this target population, not available for widespread use or based on early evaluations that may not be effective.^18^

In this paper, we have stated the non-specific and currently available treatment options for diarrhoea in LMICs to provide context. However, we aim to define a TPP for the development of novel enteric antimicrobials for the treatment of paediatric (<5 years old) diarrhoea (acute and/or severe) in LMICs. We will also discuss the relevant and limiting factors that should be considered in the development of a TPP in LMIC settings.

### Currently available treatments options for diarrhoea

The WHO Access, Watch, Reserve (AWaRe)^2^ handbook states that in most cases acute diarrhoea management of infection does not require antibiotics. This is due to the high prevalence of viral infections and the self-limiting nature of infection. The recommended standard treatment is zinc with oral rehydration and electrolyte replacement.^2^

However, a recent UNICEF report states that (despite effective development cooperation with local governments that provide water, sanitation and hygiene services to communities, the WASH initiative), 785 million people are still without access to clean water (either for drinking or sanitation).^19^ This results in a large at-risk population for severe enteric infection, but who also have better access to monotherapy standard of care (SoC) antimicrobials than clean water. For acute bloody diarrhoeal disease and in the immunocompromised, WHO recommendations are first line empirical treatment with ciprofloxacin, whereas second-line empirical treatment includes third-generation cephalosporins and azithromycin.^20^

Despite the high levels of AMR reported in LMICs, there are no unified specified standard dose, dosing frequency or duration guidelines for first- or second-line treatments of enteric pathogens.^21^ Second-line treatments using an oral cephalosporin are often regional, country and community dependent, with economic factors determining the specific beta-lactams used. The cost of enteric infection therapies, as well as the cautions and warning for the corresponding therapies, also influence treatment. These are shown in Supplementary Tables S1 and S2 (available as Supplementary data at *JAC-AMR* Online) in the Supplementary Materials.

## Attributes of a target product profile for bacterial paediatric diarrhoea

### Indications for use

The indication for minimal TPP use is based on syndromic suspected or confirmed acute/severe enteric illness including, high temperature >37.5°C (99.5°F), abdominal pain and/or diarrhoeal disease with or without blood in patients ≤5 years old in LMICs (where resource limitations may apply).^21^ The indication for preferred treatment is based on both syndromic and pathogen-directed diagnosis (where access to baseline diagnostic tools are available) due to *E. coli*, *Shigella* spp., *Salmonella* spp. or *Campylobacter* spp. in patients ≤5 years of age in LMICs.

The development of an anti-infective agent that is at least as effective as existing therapy with SoC antibiotics and is compatible with WHO recommendations is essential. Importantly, the WHO guidelines emphasize the use of oral rehydration plus zinc where possible. Therefore, antibiotics should only be used where symptoms are severe, worsening or when rehydration is not possible. These antibiotics should also be suitable for patients with diarrhoea, the immune-compromised or the severely malnourished.^21–23^

### Target population

The minimal TPP population is children under 5 years with severe bacterial diarrhoea. This is defined as either syndromic, i.e. high frequency (>6/day) bowel movements, loose/watery stools or mucus/blood-stained stools within a 24-hour period, or defined through pathogen-directed bacterial identification using rapid diagnostic tests. The target population should be based on the presence of confirmed or suspected bacterial infection with enteric *E. coli*, *Shigella* spp., *Salmonella* spp. or *Campylobacter* spp. to optimize effective and targeted treatment and reducing risk of AMR.

It is important to acknowledge that novel antibiotics must be able to be taken in parallel with local commonly available treatments such as milk (used as stomach protection)^24^ and/or medicinal plants (ginger, turmeric or garlic that are used due to their anti-inflammatory properties).^25^ This is an important consideration from a global health perspective, in regions where; antibiotic access is limited and traditional medicines are more readily available, and where the combination of multiple treatments is common practice due to cultural practices and/or cost constraints.

Preferred TPP could be extrapolated to apply to older children and adults in cases of natural disaster, civil disturbance or warfare or in regions where access to clean drinking water is not possible. Adults could also be treated in cases of severe traveller’s diarrhoea, which could provide vital safety and efficacy studies in a more stable population.

### *In vitro* potency and clinical efficacy

In the minimal TPP, agents should have *in vitro* activity against target pathogens (including antibiotic resistant isolates), no/low cross resistance to known antibiotic classes used in the treatment of paediatric diarrhoea, intracellular activity and a low propensity for mutational resistance development. Intracellular activity is considered essential as all four target pathogens can adopt an intracellular lifestyle. In addition, there is insufficient understanding of the pharmacokinetic/pharmacodynamic determinants of clinical success in acute bloody diarrhoea to base clinical breakpoints on PK/PD and establishing clinical correlates between pathogen MIC and clinical outcomes in clinical trials remains challenging. Therefore, a sensible approach would be to define *in vitro* potency relative to wild-type cut-offs. These wild-type cut-offs are designed to identify strains with phenotypically raised MICs related to a known mechanism of resistance. Therefore, in the preferred TPP, >95% strains of *E. coli Shigella* spp., *Salmonella* spp. and *Campylobacter* spp. should have an MIC below the wild-type cut-off.

For a newly developed enteric infection antibiotic, non-inferior clinical evidence and microbiological activity relative to existing SoC therapies must be demonstrated in clinical trials. Ideally, relapse rates should be low and activity demonstrated against resistant target pathogens. Any new therapy should reduce/eliminate chronic carriage in the gastrointestinal tract and eliminate persistent faecal shedding. The preventing the long-term release of pathogens in the stool would reduce the prevalence of community transmission and outbreaks. The minimal TPP efficacy endpoints are based on reduction in loose stool frequency within a 24-hour period,^23^ i.e. after treatment initiation, <6/day bowel movements, cessation of mucus/blood-stained stools after 3–5 days and/or reduction in fever ≤37.5°C, which aligns with WHO guidelines for acute diarrhoea management^20^ and FDA expectations for anti-diarrheal therapies.^26^ The preferred TPP can be based on confirmation of eradication via companion diagnostics pathogen clearance (early, sustained and long-term), and to provide epidemiological surveillance to monitor/limit secondary case infections. This could lead to longer-term clinical studies where minimal relapse rates and secondary cases are used as preferred endpoints.

It is essential that the propensity for resistance development is monitored during clinical trials. We acknowledge that with new agents’ significant levels of resistance in initial treatment clinical trials are unlikely. Unfortunately, resistance is likely to increase over time, so monitoring of new agents is required to ensure continued potency. In addition, conducting placebo-controlled studies in groups of patients where antimicrobial therapy has not previously been administered could be considered. In a preferred TPP, a companion diagnostic would be developed to ensure that correct patient populations are treated and unnecessary exposure to antibiotic therapy is avoided.

### Pharmacokinetics

The optimal pharmacokinetic properties of an antibiotic to treat bacterial diarrhoea are unknown. It is likely that high intra-luminal drug concentrations would be desirable with as little binding to faecal material as possible. It could be argued that some concentration in the bowel wall and tissues distant from the bowel lumen would help prevent translocation and secondary bloodstream infection with sepsis. For example, the non-absorbable antimicrobial rifaximin, which can be used to treat enterotoxigenic and enteroaggregative *E. coli*, is less effective against invasive bacterial pathogens such as *Shigella* spp. and *Campylobacter* spp.,^27–29^ The minimal TPP would support intracellular penetration and biliary excretion in acute infections. In addition, the preferred TPP would be available to include immunocompromised individuals, and patients with renal or hepatic insufficiency.

### Safety and tolerability

The proposed TPP must demonstrate a clinical safety comparable to SoC therapies with the same or similar dosing regimens. The therapeutic candidate should have good tolerability in children while having a minimal impact on the paediatric gastrointestinal microbiome. The ideal therapeutic should also be safe for use in immunocompromised and/or malnourished children. Manageable risks and adverse event profiles will be different within different social economic areas. We must acknowledge that benefit–risk assessments in high-income countries (HICs) and LMICs have different focal points and clinical needs. HICs have formal frameworks, are evidence based and have regulatory and policy integration. LMICs focus on feasibility. They must overcome barriers such as limited formal assessments, regulatory and policy integration often adapted from HICs, and have minimal epidemiology and cost data. This is reflected in some SoC agents recommended for widespread (hospital and community) use in LMICs, e.g. use of ciprofloxacin (fluroquinolone).^20^ WHO guidelines recommends SoC antibiotics including ciprofloxacin for enteric infections in LMICs, but in recent years specifically, community practice in HICs the use of fluoroquinolones has been limited in due to many regulators issuing several warnings about safety concerns.^30,31^ In addition, the use of ceftriaxone as a broad-spectrum intravenous agent may not be a suitable comparator for oral therapy due to the biases between route of admission, convenience/mobility of the patient and associated costs. While azithromycin may seem a suitable comparator for assessing a new agent’s adverse event profile, the high incidence of resistance may limit its use as a comparator in clinical trials in some geographies. There are many potential adverse events associated with existing SoC antimicrobials (Supplementary Figure S1). The ideal antibiotic should have manageable adverse events (caused by the drug itself) and demonstrate an appropriate benefit–risk ratio with extensive, high quality clinical and administrative data. As this newly developed antibiotic for enteric infections will be targeting pathogens which exist within the gut, it is of particular importance that any antimicrobials used do not have a detrimental effect on the gut microbiome. If this is altered resulting in reduced diversity or dysbiosis, there could be significant consequences for the patient, which include gut diseases such as *C. difficile*-associated diarrhoea and/or a predisposition to other diseases later in life.^29^ Therefore, a minimal impact on the normal microbiome is preferred.

### Formulation, dose and route of administration

Multi-particulate products such as orodispersible minitablets, oral soluble films, chewable soft-gel capsules and liquid suspension formulations, which could incorporate medicated dosing straws, should be administered orally.^32^ Injectable sterile solutions administered intravenously or intramuscularly would also be optimal for the minimal TPP. Oral formulations for the treatment of infection will facilitate with patient mobility allowing treatment to be administered outside of the hospitals, while intravenous formulations could be more appropriate for inpatient therapy of more seriously ill patients where prompt commencement of treatment is essential. Parenteral administration would also allow for treatment when oral administration is not feasible (e.g. vomiting, severe malnutrition, altered consciousness). Where clinical improvement is observed, it may be possible to de-escalate therapy i.e. switch from parenteral to oral medication. Advantages of de-escalation optimizes antibiotic use and minimizes AMR by reducing potential damage to the microbiome, selection pressure for resistance organisms and healthcare costs.

A preferred TTP would include easily swallowed oral formulations that are palatable and are acceptable to young children. Recently, there has been development of delivery systems such as the ‘nipple shield’, which, as a concept, uses milk as the vehicle for paediatric formulations improving palatability.^33^ The use of devices such as ‘pill swallowing cups’ would also aid administration if dosage were in solid forms. Smaller particulates such as ‘Sprinkles’ can be administered with food and has been shown to improve organoleptic properties and in turn increases the likelihood of compliance.^34^ Dispersible tablets or sachets combined with hydration packs^35^ in cases of natural disaster, war and/or political displacement would also be beneficial. The use of microneedle skin patches is an alternative option for administration,^36^ but we are not aware of this approach ever having been used to treat diarrhoea with an antimicrobial and it also has cost implications.

A 3-day antibiotic regimen (once a day, OD, or twice a day, BD) has been used to treat bacterial diarrhoea and would represent the ideal target. However, it is unclear whether more severely ill patients would require longer courses or more frequent dosing, and whether longer courses may result in fewer secondary cases where family members and other persons are in proximity. The minimal treatment duration should be ≤1 week, except in the most recalcitrant cases. Short courses of therapy should be used to maximize compliance, to minimize cost for the patient and/or healthcare provider, as well as minimizing adverse drug reactions and the overall impact on the patient microbiome.

### Drug interactions

As previously mentioned, negative interactions with local traditional medicine, zinc and milk should be minimal. The use of antibiotic combinations may be needed to cover all pathogens in severely ill populations and patients with diagnostic uncertainty, both pharmacological and microbiological adverse interactions should be avoided where possible. Children with HIV and TB are particularly vulnerable to severe diarrheal disease, therefore, it is essential that interactions with antiretroviral drugs and drugs commonly used for the treatment of TB are minimal.

### Stability and storage

It is vital that new antibiotics have adequate shelf life and stability i.e. heat stable at 35°C and 65% relative humidity with a 1–3-year shelf life is the minimal requirement for this TPP.^37^ The preferred TPP would have no requirement for refrigeration to facilitate distribution to all required regions. Dispersible formulations are also preferred as they are often more convenient and less costly to transport and easier to store compared with liquid formulations.

### Access and affordability

Access should be based on ethical promotion and fair distribution across LMICs, minimal infrastructure requirements (ideally using existing logistic supply chains) or implementing improved services where possible. The anti-infective must be physically robust to allow patient/medical access in extreme climates or extremely rural settings. The application of TPP must be user friendly to prevent blocked access by structural and system level barriers, aiding patient compliance. Maximal affordability should also be paired with the low cost of production targets in mind, cost should be comparable to existing SoC, details can be found in Supplementary Table S1. Ciprofloxacin tablets (pack of 10, 250 mg), azithromycin oral suspension (200 mg, 15 mL) meropenem powder of injection (500 mg, pack of 10) have price points of; $0.28, $2.96 and $31.94, respectively. Restrictions must also be in place on all new anti-infectives intended for humans and should be registered for human use only. The manufacturing site should also adhere to industry standards so that waste emissions to the environment are kept to a minimum.^38^

The preferred TTP should incorporate carefully considered access and affordability strategies early in development. Formulations should be accessible on a local and national level, and synthesized following good manufacturing practice (GMP) pathways to reduce manufacturing costs. In more recent years, we have seen examples of antimicrobials using various mechanisms such as generic patent licensing or tiered pricing.^39^ Payment models incentivising access that is de-linked from sales volume should also be considered. There should be practical strategies in place for the use of reserve antibiotic combinations/formulations,^40^ where local community leaders, pharmacies and healthcare professionals play a pivotal role in ensuring stewardship policies are adhered to. Empowering governments to conduct inspections of pharmacies and to retain prescriptions will help ensure compliance. Penalties in cases of non-compliance could also be enforced when required. Table 1 summarizes these points discussed.

**Table 1.** Minimal and Preferred TTPs for therapy targeting severe acute diarrhoeal infections, by *E. coli*, *Salmonella* spp., *Shigella* spp. and *Campylobacter* spp. in children

	Minimal TPP	Preferred TPP	Motivation
Indication for use	Paediatric diarrhoea caused by suspected or confirmed bacterial pathogen	Paediatric diarrhoea caused by suspected or diagnosed bacterial pathogen, specifically *Shigella* spp., *Salmonella* spp., *Campylobacter* spp. and *E. coli*. Acute infection or carrier status	Common pathogens associated with acute and severe paediatric diarrhoea
Target population	Children <5 years old	Older children and adults in cases of natural disaster, civil disturbance or warfare. Adults with travellers’ diarrhoea	To prevent mortality and morbidity in paediatric and adult populations
Access and affordability	See paragraph above	See paragraph above	Access and affordability should foster equality
Safety/tolerability	Clinical safety comparable to current therapies with minimal impact on the gut microbiome	Clinical safety comparable to current therapies, good tolerability in children and minimal impact on gut microbiome	Focus of tolerability must be on the patient population
*In vitro* activity	>95% of an MIC below the wild-type cut-off. *In vitro* activity against *E. coli*, *Shigella* spp., *Salmonella* spp. and *Campylobacter* spp. Low cross resistance to known antibiotics classes, intracellular activity. Low propensity for mutational resistance development	>95% of an MIC below the wild-type cut-off. *In vitro* activity against *E. coli*, *Shigella* spp., *Salmonella* spp. and *Campylobacte*r spp. Low cross resistance to known antibiotics classes. Intracellular activity. Low propensity for mutational resistance development	Should have a broad spectrum of activity against bacterial gastrointestinal pathogens associated with paediatric and adult diarrhoea
Clinical efficacy	Non-inferior clinical activity against bacterial enteric pathogens compared to current SoC antibiotics in susceptible strains and a low relapse rate. Clinical activity in infections due to pathogens resistant to current therapies. Not using companion diagnostics as endpoints	Non-inferior clinical activity against bacterial enteric pathogens compared to current SoC antibiotics in susceptible strains and a low relapse rate. Prevent convalescent faecal shedding. Clinical activity against infections caused by pathogens resistant to current therapies. Use of companion diagnostics as endpoints	To be superior to currently available therapies
Formulation/Route of administration	Multipartiulate products; Orodispersible minitablets, oral soluble films, chewable soft-gel capsules, Oral liquid suspension, oral + parenteral administration. Easy to administer to children including medicating dosing straws	Multipartiulate products; Orodispersible minitablets, oral soluble films, chewable soft-gel capsules, Oral liquid suspension, oral + parenteral administration. Easy to administer to children including formulations which improve palatability i.e. nipple shield, medicating dosing straws, pill swallowing cups, and sprinkles. Dispersible tablets or sachets combined with hydration packs	Based on appropriateness for target population; water access and compliance
Dose regimen	1–3× daily, duration up to 5 days	1–2× daily, duration up to 3–5 days	Short dosing regimen and limited daily administration to maximize patient compliance
Product stability and storage	Heat stable. 3 year shelf life in hot and humid conditions (35°C and 65% relative humidity)	Heat stable. 3 year shelf life in hot humid conditions (35°C and 65% relative humidity). Stable formulated suspension (multiple days) without refrigeration	To facilitate easy use and storage in LMICs
Pharmacokinetics	Pharmacokinetic data available to support use in acute infections, intracellular penetration and biliary excretion	Pharmacokinetic data available to support use in acute infections including children <5 years, older patients >65 years old. Support use in immunocompromised patients, patients with renal or hepatic insufficiency, intracellular penetration and biliary excretion	To ensure effectiveness in the target population

## Considerations while developing a TPP for low- and middle-income countries

The high burden of AMR, the lack of antibiotic development within some LMICs combined with well documented antibiotic market failures, contribute to the current AMR and access crisis.^6^ Difficulties in accessing data, policy, limited capacity, limited infrastructure and, perhaps most importantly, engagements with patients and the broader community exacerbate the challenges in research and development and equitable access of novel antibiotics. A TPP must begin and end with the patient. As we explore the key factors for a successful drug development in LMICs, our focus should remain firmly on patient benefit while acknowledging the significant gaps that must be bridged.

### Data available in LMICS

There are limited data available for LMICs. However, using mortality data,^41^ significant variations emerge in disease epidemiology between HICs and LIMCs underpinned by resources, funding, healthcare structures and population density. More than 80% of the world’s population live in densely populated settings within LMICs. These settings disproportionately bear the highest burden of AMR.^41^ When assessing global AMR from an HIC perspective, some bias can be encountered, therefore, the unmet need in the most vulnerable populations should guide our work.

While there are many global initiatives to assess the global burden of AMR through surveillance and antimicrobial susceptibility testing, most do not include isolates specifically from enteric infections. These schemes can capture data from enteric pathogens detected in the bloodstream but are not necessarily representative of the high incidence of bacterial enteric infection reported in LMICs. In addition, *Shigella* spp. and enteric *E. coli* are rarely isolated from blood cultures and as a result, the aetiology of bacterial enteric infections from LMICs are largely unknown.^42^

A second influencing factor is the lack of diagnostic microbiology laboratories for enteric pathogens in LMICs, resulting in paucity of baseline isolation and resistance data.^43^

Furthermore, the published datasets available such as Global Enteric Multicentre Study indicates bias in sampling centres such as public versus private hospitals, central versus regional centres and community healthcare versus hospital intensive care unit. All this generates a picture that is not representative of the whole burden of disease.^6^ To close the knowledge gap, we require more robust surveillance systems to obtain accurate data on the incidence of enteric infection across LMICs. We hope that some schemes like WHONet,^44^ and ASLM^45^ will provide robust mechanisms of data collection capacity on the incidence of bacterial infections so that the burden of resistance in key pathogens can be established and monitored over time. This could help inform prescribing policies and treatment guidelines.

### Lack of paediatric-specific data

Considerations for paediatric product development plans are typically neglected due to perceived small market size and return of investment, for example, HICs <1 death per 100 000 population. A small addressable market combined with access to alternative treatments options directly affects investment in collection of paediatric-specific data within HICs. Despite the high disease burden (Sub-Suharan Africa^1^ 160 deaths per 100 000 population), purchasing power (relatively low GDP per capita LMIC in comparison with HIC), extremes in healthcare infrastructures, regulatory uncertainties, competitive priorities (HIV, TB), regulatory pathways (which are often fragmented into national agencies) and investment incentives all prevent collection of comprehensive paediatric-specific datasets. This highlights a stark reality; The higher the mortality rates the lower the incentive for innovation as commercial appeal is less, directly affecting the most vulnerable population.

The lack of paediatric data, combined with a lack of current SoC paediatric guidelines enforces the previously stated paradox. It is widely documented that resistance rates are significantly elevated in LMICs, but the quantification of these rates and scale of the unmet needs are largely unknown. This lack of data encourages the continuation of overuse of antibiotics that perpetuates rising AMR.

Building a dedicated surveillance network could provide precise disease burden data within this population, which could be used to inform clinical trial designs. The designing of bespoke paediatric clinical trials could highlight the clinical need and potentially offer improved financial incentives for developers.

The mandate for a robust paediatric surveillance framework is clear; however, we must accept that the commercial disincentive, disease burden and developmental challenges all contribute to an inherently protracted process. Overcoming these barriers requires sustained multi-national dedication, long-term governmental/capital commitment and cross-sector alignment.

### Access to clean water and sanitation

Lack of access to clean water and sanitations is the second biggest factor affecting AMR rates in LMICs.^19^ This is important to consider when designing a TPP because WASH implementation is not easily or uniformly addressed across LMICs.^20,46^ Poor sanitation and hygiene with limited access to clean water often leads to the spread of enteric disease and/or reinfection by enteric pathogens. In cases of mass population displacement due to natural disaster, civil disturbance or warfare, the lack of access to basic water facilities leads to an increase in antibiotic use and misuse. Poor facility management polices lead to inadequate or damaged infrastructure. This poses significant risks of environmental contamination with antibiotic residues, ultimately leading to increased levels of AMR.

### Polymicrobial infections

Unfortunately, enteric disease is not limited to bacterial infection. The high mortality in children under 5 years old is primarily a result of infection with viral pathogens such as rotavirus/adenovirus.^4^ There is also high prevalence of parasitic disease such as *Entamoeba* spp., *Cryptosporidium* spp. and *Giardia* spp. infection, from all pathogens related to enteric disease, can arise due to frequent exposure (and re-exposure within the same household) aggravated by prolonged shedding. Infections from multiple enteric pathogens can be observed in a single host due to polymicrobial carriage (although this is poorly understood).^47^ Polymicrobial infection could have an impact on the severity of enteric bacterial diseases and mortality. It can also confound diagnostics, as the detection of one pathogen does not exclude the presence of others. Making the treatment of such infections more complex in resource-limited settings. We should encourage a multifaceted approach, encompassing all treatment options such as new antimicrobials, vaccines, antibiotic combination therapies and novel therapies such as bacteriophage.

### Co-morbidities

Within some LMICS, there are reportedly high rates of tuberculosis and HIV infection leaving a proportion of the population immunocompromised (including children).^48–51^ Increased use of antimicrobials for the treatment of tuberculosis and sexually transmitted infections such as rifampicin, levofloxacin, amikacin and azithromycin, Doxycycline respectively may lead to an increased antimicrobial usage within a population. Which potentially could increase the AMR observed in enteric pathogens. There are also overlaps in clinical presentation/symptoms. For example, gastrointestinal tuberculosis albeit relatively uncommon leads to diarrhoea, and can complicate the diagnosis and therefore treatment. There is also overlap between HIV and diarrheal diseases as co-infection is common, occurring in 90% of HIV infected children.^48,52^ Efforts to confront both diarrhoeal diseases and other co-morbidities must be addressed via healthcare infrastructure, improving sanitation and hygiene as well as ensuring access to appropriate treatments.

### Access to therapeutics

The pharmaceutical regulatory landscape often faces gigantean challenges relating to the access of safe, effective and affordable medicines while navigating systemic challenges in both HICs and LMICs. Pharmaceutical sectors and regulatory within HICs have specific challenges around slow approval processes, high labour and operational costs and pharmacovigilance enhancements. However, there appears to be more homogeny in antibiotic access.

**Figure 1.**
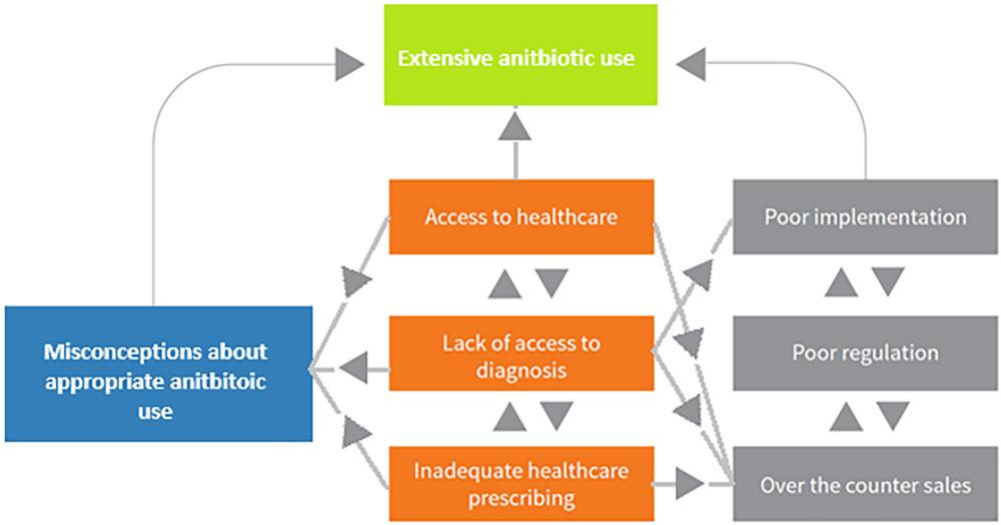
Factors associated with extensive antimicrobial use in LMICs.

Conversely LMICs have significantly different healthcare needs, economic resources and regulatory capacities. This makes access inconsistent and disproportional which results in common over-the-counter practices, leading to inappropriate and extensive antibiotic use. Instances of this include inconsistent regulation of antibiotic sales, inadequate healthcare prescribing based on cost rather than efficacy, limited use of diagnostic tests or lack of access to health systems entirely and often a general misconception that antibiotics should be used in the first instance of acute diarrhoea.^53^ Initiatives such as those of the African Medicines Agency^54^ have described some of the existing factors associated with extensive antibiotic use in LMICs. Figure 1 depicts the complexity in system level factors, behavioural factors and healthcare delivery issues, which influences healthcare paths often resulting in an unclear organizational route. These factors must be acknowledged and considered when assessing the best way to introduce a new therapeutic.

Regulation may address some of these issues, and there are examples where LMICs have implemented measures such as banning over-the-counter antibiotic sales. However, such actions have sometimes led to unintended consequences including increased circulation of counterfeit or substandard alternatives calling for stringent market control.^55^ Such an approach should be thoughtfully integrated into national health policies.

### Substandard and counterfeit antibiotics in LMICs

Medicines manufactured locally often do not meet the quality standards set by the local regulatory agencies.^48^ This does not include counterfeit medicines but is referring to poor-quality active pharmaceutical ingredients (APIs) used to make finished pharmaceutical products (FPPs).^56^ FPPs in LMICs are monitored, however, the poor-quality APIs which are cheaper and are often left unregulated are used extensively. The poor-quality API is often transferred to the FPP and can result in poor drug stability and efficacy.^57,58^ There are also concerns of contamination during production and are found in local manufacture where GMPs are not followed, leading to cross-contamination among drugs produced in the same facility.^59^ Some LMICs have robust mechanisms in place ensuring quality, safety and efficacy, while other LMICs struggle with resource constraints resulting in considerable disparities in healthcare between nations.^60^

Counterfeit antibiotics are deliberately mislabelled with the purpose of deceiving consumers. These may contain no active components or even harmful substances that often yield a hidden and sometimes fatal outcome. Overall, use of substandard and/or counterfeit antibiotics leads to a mistrust in healthcare systems and an increase in AMR. Improved medicine quality surveillance, regulatory capacity, enforcement and education could begin to address these challenges.

### Non-human antimicrobial use

In LMICs, human intended antimicrobials are commonly used for non-human applications such as agricultural/livestock/meat processing. Sometimes this is done indiscriminately, even if individual governments have legislations stating otherwise.^61^ Non-human uses based on economics rather than stewardship may increase the risk of AMR burden significantly. The proximity of animals and humans also makes the spread of resistant bacteria from animals to humans (and vice versa) more likely. Therefore, in an ideal TPP and in practice, the new anti-infectives for human use should remain for human use only.

### Treatment endpoints

In HICs, diagnosis-based treatment is preferred. Unfortunately, this is not a reality in LMICs where most healthcare infections are managed on the basis of syndrome. Low-cost, rapid, point-of-care diagnostics are encouraged to optimize patient management and to reduce the overall cost of treatment. Or, if specific diagnoses are not possible, application of tests that can differentiate between viral or bacterial infections at the point of care would be preferable. Where this is not feasible, the use of a Medical Avatar (virtual training environment) designed to educate on a specific application or to advise on the appropriate antibiotic therapy, duration and/or stewardship should be applied.^62,63^ General advice could be accessed on smartphones, which could help change local practices and contribute to an increase in patient AMR awareness. A new algorithm (ALMANACH) for managing childhood illness using smartphone technology in Tanzania reduced antibiotic prescribing by 80%.^62^ It is important to state that this technology is not without drawbacks. While algorithms are continually improving and biomarkers are being identified for the use in surveillance of diarrhoeal diseases, differentiating between bacterial and non-bacterial infections remains problematic. Complications, such as reinfection within one household, are limitations and this type of work still has a relatively high cost.^63^

### Conclusions for successful TTP in LMICs

This TPP outlines the preferred and minimal characteristics required for an anti-infective with a primary focus of enteric pathogens causing diarrhoeal disease within a LMIC paediatric population. This TPP could be operationalized in drug development pipelines where early alignment and clear goals are critical to the effective progression of a successful candidate. All Go/No Go decisions should directly reference the TPP endpoints stated above in pre-clinical and clinical trial phases. TPP endpoints are also applicable to refine patient stratification when investigating companion diagnostics and data generated provides evidence for patient-reported outcomes and health economics data. Diagnostic tools must be both sustainable in low-resource settings and scalable on a global scale, therefore there must be consolidation and balance with scientific, commercial and regulatory risk. However, there are also many critical factors that must be addressed to enable successful and impactful therapy.

Infrastructure policies (which are often variable and face combinations of financial, technical, institutional and socioeconomic barriers), natural disasters (which should be improved by management boards and polices) and civil disturbances affecting access to clean water are largely beyond our control. We can and should address other critical gaps through clear communication and targeted awareness raising campaigns. It is the concretion of interventions beginning in the community which will facilitate and sustain engagement with patients, medicine dispensers, healthcare professionals and policy makers.

It is important to acknowledge the reluctance among healthcare professionals to adopt new prescribing guidelines, particularly in low-resource settings where the burden of critically ill patients is high. Similarly, local communities may be hesitant to change established treatment practice, especially when economics or cultural factors play a central role. To address these challenges, continuous education is essential for healthcare providers, and awareness campaigns focused on patients and caregivers can help change attitudes towards appropriate antibiotic use. Medicine dispensers should receive targeted training to promote best practices across sectors. Policy makers play a critical role in eliminating the circulation of spurious and falsified distribution. Investment in robust monitoring systems for market control, able to detect spurious and falsified medicine, including an API source is needed, with appropriate penalties in place. Finally, expanding surveillance capacity and data collection in LMICs, specifically for paediatric patients, will inform future stewardship strategies and support awareness raising, prescribing practice, responsible antibiotic use and inform policy development.

Today 137 counties^64,65^ are classified as LMICs, spanning a huge geographical area and containing diverse cultural practices.^57^ Variation between prescription practices, access, adherence and attitudes vary between continents, countries and, often, regionally and locally.^66–70^ We must achieve sustained engagement from patients, prescribers, local communities, policy makers, governments and global initiatives to mitigate the burden of AMR and of diarrheal infections in children. With a goal to make these entirely preventable mortalities a thing of the past.

## Supplementary Material

dlag128_Supplementary_Data

## Transparency declarations

M.A. holds grants/activities with Merck, Shionogi, InfectoPharm, GSK, Roche, Bioversys, Venatorx Pharmaceuticals, Tecnimede group, Santero and Advanz Pharma. D.K.M. is an employee of Bioaster and works voluntarily for INCATE. J.H. receives funding from the Combating Antibiotic Resistant Bactria Accelerator (CARB-X), a government-supported not-for-profit organization. T.C., E.D.J., V.G., L.A.H., S.M.K., S.O.B., K.M.O., D.L.P. and D.Y.M. have no conflicts of interest. A.P.M. holds research grants/activities with Merck, Shionogi, InfectoPharm, GSK, Roche, Bioversys, Venatorx Pharmaceuticals and NIHR.

## Supplementary data

Figure S1 and Tables S1 and S2 are available as Supplementary data at *JAC-AMR* Online.
