# Supplementary material for: Defining a target product profile for antimicrobials to treat severe paediatric bacterial enteric infections in low- and medium-income countries, caused by E. coli, Shigella, Salmonella and Campylobacter species
Source: JAC Antimicrob Resist. 2026 Jul 10;8(4):dlag128. doi: 10.1093/jacamr/dlag128 (PMC13351170; doi:10.1093/jacamr/dlag128)
Supplement: dlag128_Supplementary_Data [file dlag128_supplementary_data.docx]

**Supplementary material**

Figure S1 – Diarrhoeal mortality rates per 100, 000 and counts by broad age categories A, and under 5 years groups B, from 1990-2021 ^56^


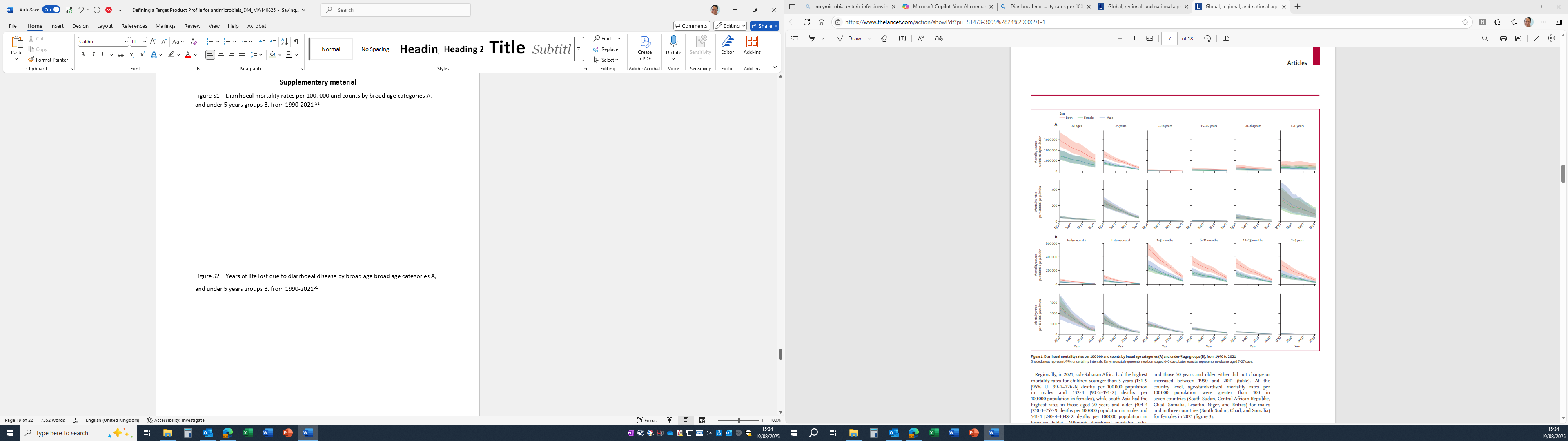


Figure S2 – Years of life lost due to diarrhoeal disease by broad age broad age categories A,

and under 5 years groups B, from 1990-2021^56^


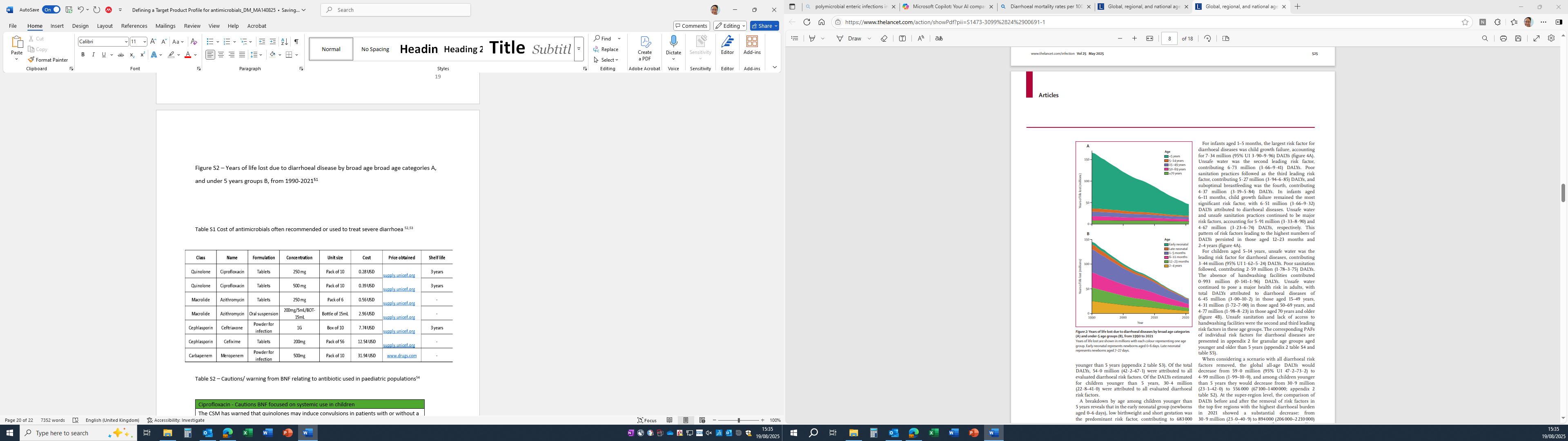


Table S1 Cost of antimicrobials often recommended or used to treat severe diarrhoea ^59,60^

| **Class** | **Name** | **Formulation** | **Concentration** | **Unit size** | **Cost** | **Price obtained** | **Shelf life** |
| --- | --- | --- | --- | --- | --- | --- | --- |
| Quinolone | Ciprofloxacin | Tablets | 250 mg | Pack of 10 | 0.28 USD | [supply.unicef.org](https://supply.unicef.org/catalogsearch/result/?q=mer) | 3 years |
| Quinolone | Ciprofloxacin | Tablets | 500 mg | Pack of 10 | 0.39 USD | [supply.unicef.org](https://supply.unicef.org/catalogsearch/result/?q=mer) | 3 years |
| Macrolide | Azithromycin | Tablets | 250 mg | Pack of 6 | 0.56 USD | [supply.unicef.org](https://supply.unicef.org/catalogsearch/result/?q=mer) | - |
| Macrolide | Azithromycin | Oral suspension | 200mg/5mL/BOT-15mL | Bottle of 15mL | 2.96 USD | [supply.unicef.org](https://supply.unicef.org/catalogsearch/result/?q=mer) | - |
| Cephalosporin | Ceftriaxone | Powder for infection | 1G | Box of 10 | 7.74 USD | [supply.unicef.org](https://supply.unicef.org/catalogsearch/result/?q=mer) | 3 years |
| Cephalosporin | Cefixime | Tablets | 200mg | Pack of 56 | 12.54 USD | [supply.unicef.org](https://supply.unicef.org/catalogsearch/result/?q=mer) | - |
| Carbapenem | Meropenem | Powder for infection | 500mg | Pack of 10 | 31.94 USD | [www.drugs.com](http://www.drugs.com/) | - |

Table S2 – Cautions/ warning from BNF relating to antibiotic used in paediatric populations^61^

| **Ciprofloxacin - Cautions BNF focused on systemic use in children** |  |
| --- | --- |
| The CSM has warned that quinolones may induce convulsions in patients with or without a history of convulsions; taking NSAIDs at the same time may also induce them. |  |
| Ciprofloxacin is used for the treatment of disseminated gonococcal infection, but is not licensed for this indication. |  |
| Tendon damage: |  |
| Tendon damage (including rupture) has been reported rarely in patients receiving quinolones. Tendon rupture may occur within 48 hours of starting treatment; cases have also been reported several months after stopping a quinolone. Healthcare professionals are reminded that: |  |
|  |  |
| quinolones are contra-indicated in patients with a history of tendon disorders related to quinolone use; |  |
| the risk of tendon damage is increased by the concomitant use of corticosteroids; |  |
| if tendinitis is suspected, the quinolone should be discontinued immediately. |  |
| MHRA/CHM advice: Systemic and inhaled fluoroquinolones: small increased risk of aortic aneurysm and dissection; advice for prescribing in high-risk patients (November 2018) |  |
|  |  |
| MHRA/CHM advice: Fluoroquinolone antibiotics: new restrictions and precautions for use due to very rare reports of disabling and potentially long-lasting or irreversible side effects (August 2023) |  |
